# Assessment of probiotic strain *Lactobacillus acidophilus* LB supplementation as adjunctive management of attention-deficit hyperactivity disorder in children and adolescents: a randomized controlled clinical trial

**DOI:** 10.1186/s12888-023-05324-4

**Published:** 2023-11-09

**Authors:** Reham M. Elhossiny, Heba H. Elshahawy, Hanan M. Mohamed, Reham I. Abdelmageed

**Affiliations:** 1 Pediatrics Department, Faculty of Medicine, Ain Shams University, Abbassya Square, Cairo, Egypt; 2 Department of Neuropsychiatry, Faculty of Medicine, Okasha Institue of Psychiatry, Ain Shams University, Cairo, Egypt

**Keywords:** Attention-deficit/hyperactivity disorder, ADHD, Children, Adolescents, Probiotic, *Lactobacillus acidophilus* LB, Cognition

## Abstract

**Background:**

This study was designed to examine the possible efficacy of the probiotic strain *Lactobacillus acidophilus* LB (Lacteol Fort) on attention-deficit/hyperactivity disorder (ADHD) symptomatology and evaluate its influence on cognition function.

**Methods:**

In this randomized controlled trial, 80 children and adolescents with ADHD diagnosis, aged 6–16 years, were included. The participants were randomly assigned to two groups: one group received probiotics plus atomoxetine, whereas the other group received atomoxetine only. ADHD symptomatology was assessed using the Conners Parent Rating Scale–Revised Long Version (CPRS-R-L) and Child Behavioral Checklist (CBCL/6–18). The participants were evaluated for their vigilance and executive function using Conner’s Continuous Performance Test (CPT) and Wisconsin Card Sort Test (WCST). Both groups were assessed at the beginning of the study and the end of the twelve weeks.

**Results:**

The probiotic group comprised 36 patients, whereas the control group comprised 40 patients in the final analysis after four patients dropped out of the trial. After 3 months of probiotic supplementation, a significant improvement in the CPRS-R-L and CBCL total T scores was observed compared with those in the control group (*p* = 0.032, 0.024, respectively). Additionally, the probiotic group demonstrated improved focus attention (target accuracy rate and omission errors;*p* = 0.02, 0.043, respectively) compared with the control group. An analysis of the Wisconsin Card Sorting Test (WCST) performance demonstrated that the probiotic group had significantly lower perseverative (*p* = 0.017) and non-perseverative errors (*p* = 0.044) but no significant differences compared to the control group.

**Conclusion:**

*Lactobacillus acidophilus* LB supplementation combined with atomoxetine for 3 months had a beneficial impact on ADHD symptomology and a favorable influence on cognitive performance. As a result, the efficacy of probiotics as an adjunctive treatment for managing ADHD may be promising.

**Trial registration:**

ClinicalTrials.gov (identifier: NCT04167995). Registration date: 19–11-2019.

## Introduction

Attention-deficit/hyperactivity disorder (ADHD) is the most common early-onset neurodevelopmental disorder in the pediatric population, affecting 7.2% of school-age children globally. It is characterized by deficits in the cognitive functioning pattern, with hyperactivity, impulsivity, and attention problems that are developmentally inappropriate and significantly impairing symptoms [1]. The pathophysiological mechanism that underlies ADHD remains being investigated. Nevertheless, research highlights the complex interplay of genetic and environmental risk factors, which may underlie the observed clinical symptom heterogeneity among individuals with ADHD [2]. It has been determined that neurotransmitter dysregulation, particularly norepinephrine, serotonin, and dopamine, may play a fundamental role in ADHD pathogenesis [3]. This is supported by evidence of the abnormal gene expression linked to those neurotransmitters in children with ADHD [4]. Therefore, targeting monoaminergic systems underpins most ADHD treatments [5].

Recently, a growing interest has been directed to the bidirectional pathway between the gut and brain—the gut–brain axis (GBA). Alterations and imbalances in the gut microbiota may play a role in developing and progressing neurodevelopmental disorders like ADHD. [6, 7]. Mounting evidence hypothesized that through this pathway, neurotransmitters released by the bacteria in the intestinal lumen may stimulate epithelial cells to release hormones and cytokines. These substances may then modify neural circuitry within the enteric nervous system, thus regulating brain activity and behavior [8]. Related to this, brain diseases, such as ADHD, caused by neurotransmitter dysregulation may benefit from targeting the gut microbiota as a therapeutic approach. Detecting abnormalities in the functioning of the gastrointestinal system, altered composition in gut microbes, and an increased prevalence of inflammatory problems in children with ADHD provided further support for the involvement of the GBA in the etiology of ADHD [9–11].

Furthermore, several reports showed an association between ADHD and low levels of brain-derived neurotrophic factor (BDNF), which is essential for neuronal development, suggesting that BDNF contributes to its pathophysiology [12–14]. There is clear evidence that short-chain fatty acids (SCFAs) produced during microbial fermentation have been positively correlated with BDNF levels [12]. This suggests that the gut microbiota indirectly impacts BDNF levels, which could be modified to benefit those with ADHD [15].

Probiotics are bacteria that benefit the host body [16]. Because probiotics have various health benefits for the host, high intestinal adhesion abilities [17], and few adverse effects, they are widely applied as dietary supplements. The research results are varied, investigating the potential benefits of probiotic supplementation in ADHD regarding their type, dose, and duration and the different assessment methods throughout conducted trials. Interestingly, it has been demonstrated that probiotics could reduce the risk of later neurodevelopmental disorders in children supplemented with *Lactobacillus rhamnosus* GG (LGG) early in life [18]. *Lactobacillus rhamnosus* and *Bifidobacterium* species are the most researched strains, and their neurobehavioral impacts have been reported [19, 20]. Another probiotic bacterium, *Lactobacillus acidophilus* LB, reduces cholesterol levels and has physiological and pharmaceutical benefits in preventing and treating certain disorders [21]. However, the potential therapeutic benefits of the *Lactobacillus acidophilus* strain in children with ADHD have not yet been investigated. Accordingly, the primary objective of this randomized controlled trial was to examine the potential effects of *Lactobacillus acidophilus* LB supplementation combined with atomoxetine on the core clinical symptoms of ADHD. The secondary objective was to investigate whether 12-week probiotic supplementation could improve cognitive functions in children with ADHD.

### Patients and methods

#### Study design

This study was a 12-week randomized controlled trial set as a prospective, parallel, open-label study conducted from June 2020 to October 2021 on pediatric and adolescent outpatients with ADHD. The trial was registered at ClinicalTrials.gov (identifier: NCT04167995, on 19/11/2019). The study’s reporting complies with the Consolidated Standards of Reporting Trials 2010 statement [22]. The study was approved by the Ethics Committee of Ain-Shams University Hospitals (Ethical Committee No. FMASU 158) and was conducted following the Helsinki Declaration of 1975. The legal guardians of the participants signed the informed consent form after being provided with a thorough explanation of the procedures, the study’s purpose, and assurances of confidentiality.

#### Participants

Eighty children and adolescents aged 6–16 were recruited from the Developmental and Behavioral Pediatrics Clinic Children’s Hospital and Psychiatry Institute, Faculty of Medicine, Ain-Shams University, Cairo, Egypt. The participants fulfilled the diagnostic criteria for ADHD according to the Diagnostic and Statistical Manual of Mental Disorders (DSM-5) [23] criteria and Conner’s Parent Rating Scales-Revised (CRS-R), which a psychiatrist established before the study. The exclusion criteria were as follows: (a) individuals with an estimated intelligence quotient (IQ) less than 80% based on the Arabic version of the Wechsler Intelligence Scale for Children (WISC-III) [24]; (b) those with any significant medical or other neurodevelopmental disorders, such as autism; (c) those who have received medications for ADHD less than 8 weeks before the study; and (d) those who have taken antibiotics or probiotics recently.

#### Randomization

A statistician, independent from the study investigators, randomly allocated the enrolled participants into the probiotic group (*n* = 40) or the control group (*n* = 40) using a random number generator from a computer-based randomization software. The allocation was concealed using opaque, sealed, sequentially numbered envelopes. After obtaining informed consent, the opaque sealed envelopes were unwrapped, and the participants were enrolled in the relevant group. Only the researchers collecting and analyzing the data were blinded to the study groups (assessor-blinded).

### Intervention

The participants with ADHD randomized to the study (Probiotic) group received a probiotic preparation (Lacteol Fort®; lyophilized heat-killed *Lactobacillus acidophilus* LB*,* sachets containing 10 billion colony-forming units, manufactured by Rameda Pharmaceutical Company, Egypt, under the license of Axcan Pharma S.A, France) in a dose of two sachets disintegrated in 50-mL freshwater, twice daily, from the first day of the study until 3 months. Additionally, each parent received a daily text message reminding them to let their children take their supplements as directed and to report any side effects. The control (no probiotic) group did not receive any probiotics. All participants were on a stable pharmacological treatment for ADHD (atomoxetine) with a consistent dosage throughout the study (1.2 mg/kg/day).

### Instruments and measures

*Conners Parent Rating Scale-Revised Long Version (CPRS-R-L)* [25] *(Arabic version* [26]*).*

The CPRS-R-L is an 80-item behavior rating scale that identifies children at risk of ADHD and assesses the severity of their ADHD symptoms. On a 4-point Likert scale, from 0 to 3, parents’ responses to their children’s behavior over the previous month were rated, with 0 indicating “not at all” and scores of 1–3 indicating “just a little” to “severely affected,” respectively. The raw scores were interpreted using T-values, with scores above 60 considered moderately elevated and those above 70 considered significantly high. The scale included seven subscales, three DSM-IV Symptom Indices, an ADHD Index, and three Conners’ Global Indices. Our study targeted the three DSM-IV subscales (Inattentive, Hyperactive/impulsive, and Total score).

### Child Behavioral Checklist (CBCL/6–18)

The CBCL/school-aged (6–18 years) is a parent-rated questionnaire containing 113 items subdivided into three dimensions, noted as internalizing, externalizing, and total behavior problems, quantitatively assessing and providing dimensional insights concerning children’s psychopathology and behavioral functioning [27]. Responses to the CBCL were rated on a 3-point rating scale, from 0 to 2, with 0 indicating “not true” and scores of 1–2 indicating “somewhat” or “sometimes true” to “very true” or “often true,” respectively. Test results were interpreted using T-values, and children with scores ≥ 65 are more likely to have behavior problems with clinical relevance. The assessment was made using the Arabic version of the *CBCL/6–18*, provided by the Achenbach System of Empirically Based Assessment Foundation (Burlington, USA) after signing a license agreement with them.

### *Psychology Experiment Building Language (PEBL) version 2.0 of the Conners Continuous Performance Test (CPT)* [28, 29]

PEBL version 2.0 of the CPT measures sustained attention and impulsivity in a 14-min computerized task. Participants are instructed to hit the button whenever any alphabet letter other than the X letter is shown. The test measures selective inattentiveness (missing target stimuli: omission errors), impulsivity (false responding to non-target stimuli: commission errors), and sustained attention (reaction time and reaction time variability).

### *PEBL version 2.0 of the Wisconsin (Berg) Card Sort Test (WCST)* [30]

PEBL version 2.0 of the WCST measures executive functioning, cognitive flexibility, and set-shifting abilities. It comprises two sets of cards: 64 reaction cards and four stimulus cards. Based on the patterns present on the cards, participants were instructed to categorize them. The rule for properly sorting the stimuli shifts regularly, and the ability to change strategies that vary according to the stimuli's color, number, or shape is recorded. The participant should first choose the proper sorting principle and stick with it throughout the test to perform successfully. Shifting the matching rule to another category occurs after ten successively correct matches in one category (e.g., matching numbers). The main outcome parameters are the correct responses, categories completed, perseverative errors, non-perseverative errors, total errors, and failure to maintain a set. Executive dysfunction is assumed to be reflected in preservative and non-preservative errors [31].

### Primary and secondary outcomes

Both groups were assessed at baseline and follow-up at twelve weeks. The primary outcomes were changes in the severity of ADHD symptoms and associated behavioral problems assessed using the CPRS-R-L (Inattentive, Hyperactive/impulsive, and Total score) and CBCL (Syndrome scale and Total score), respectively. Secondary outcomes were improvements in sustained and focused attention, impulsivity, executive functioning, and set-shifting abilities based on the CPT and WSCT tasks.

### Statistical analysis

Using G*Power, the alpha error and study power were set at 5% and 80%, respectively. Assuming an effect size of 0.7 (Cohen’s d), a sample size of 40 cases per group was required, considering a dropout rate of 20%.

The collected data were revised, coded, tabulated, and introduced to a personal computer using Statistical Package for the Social Sciences, version 25.

Student’s t-test was used to evaluate the statistical significance of the mean difference between the two study groups. The chi-square test was used to compare the two study groups. The *Mann–Whitney U-test* was used to assess the statistical significance of the difference in baseline and follow-up changes between the two study groups. The Wilcoxon signed-rank test was applied to evaluate the statistical significance of the difference in scores measured twice for the same group.

## Results

**Fig. 1:**
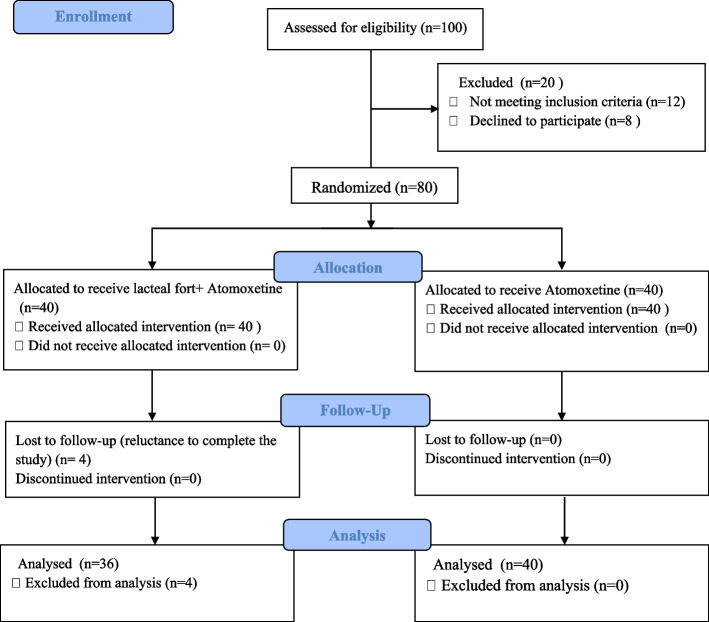
CONSORT flow chart of the recruited participants through the trial

Of 100 children and adolescents, 80 (40 in each group) met the eligibility criteria and were willing to participate in the study. However, only 36 of the 40 probiotic group participants completed the study as we failed to follow up with four children who were reluctant to continue the investigation (Fig. 1).

As shown in Table 1, the mean ages did not differ between the two groups (8.72 ± 2 years in the probiotics group and 8.48 ± 1.5 years in the control group; p = 0.543), and most participants were males (24 in the probiotics group and 27 in the control group; *p* = 0.938). Moreover, no significant difference in the IQ measurements was observed between the two groups.

**Table 1:** General characteristics of the participants

		**Group**		**Test of significance**	
		**Probiotics *(N* = *36)***	**Control *(N* = *40)***	**Value**	***P*-value**
Age, year, mean (SD)		8.72 ± 2	8.48 ± 1.5	*t* = *-*0.611	0.543
BMI, mean (SD)		19.28 ± 1	19.84 ± 1.74	*t* = 1.68	0.096
Sex, N (%)	Male	24 (66.7)	27 (67.5)	*X*^*2*^ = 0.006	0.938
	Female	12(33.3)	13 (32.5)		
IQ, mean (SD)		91.6 ± 9.65	89.68 ± 6.52	*t* = -1.019	0.311

^*^p < 0.05; Student t-test of significance (t). Chi-Square test of significance (X^2^)

*SD* Standard deviation, *N* Number

Primary outcomes.

**Fig. 2:**
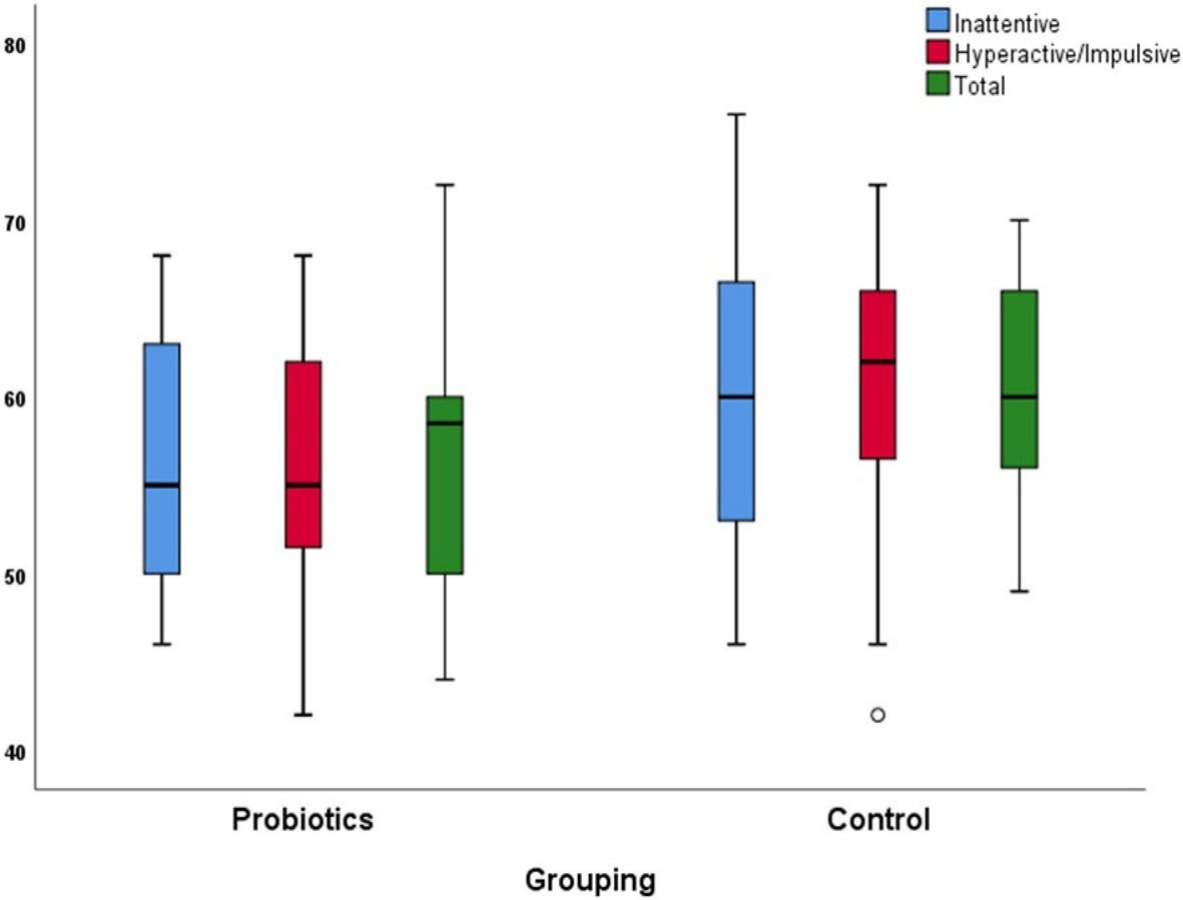
Box plot of the two study groups' Conners Parent Rating Scale T scores at follow-up measurements

**Fig. 3:**
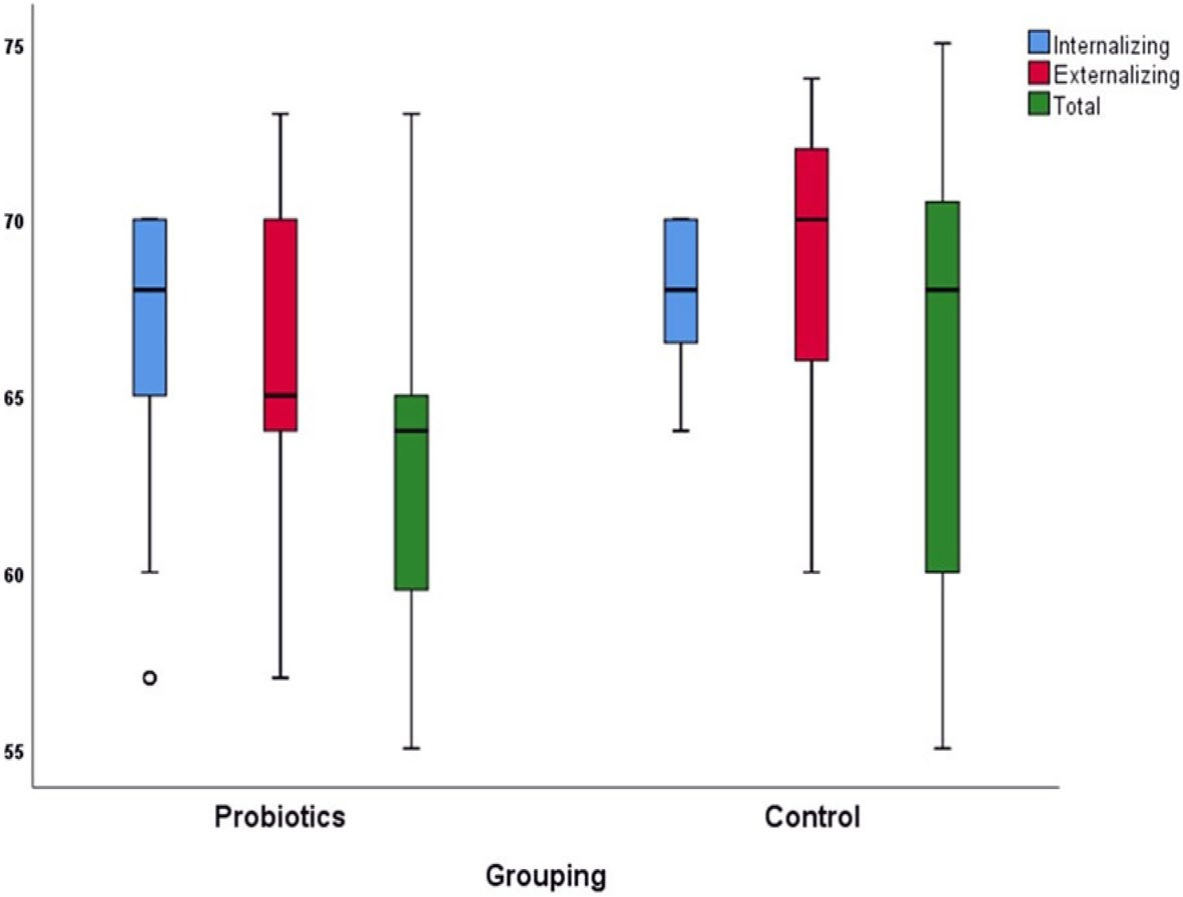
Box plot of the two study groups' Child Behavioral Checklist T scores at follow-up measurements

From baseline to 12 weeks at the end of the trial, the probiotics group showed a reduction in CPRS-R-L scores relative to the control group. Significant differences in the mean changes in the CPRS-R-L subscale T scores, DSM Inattentive, DSM hyperactive-impulsive, and DSM Total (-6.7 ± 10, -6.8 ± 8.7, and -6.11 ± 8, respectively, *vs.* -2.5 ± 6.7, -2.6 ± 12, and -2.5 ± 9.5; *p* < 0.001) were observed over 12 weeks (Table 2, Fig. 2). Similarly, the probiotics group showed a considerable improvement in the overall behavioral problems as measured using the CBCL over 12 weeks compared with the control group, with significant mean change differences in the CBCL subscale T scores on the syndrome scale internalizing, externalizing, and the total score; *p* = 0.001 (Table 3, Fig. 3).

**Table 2:** Conners Parent Rating Scale, comparing the probiotic and control group results at baseline and follow-up measurements

CPRS-R-L (Raw score)	**Probiotics *(N* = *36)***					**Control *(N* = *40)***					***p*- Value**^**(M)**^
	**Before**	**After**	**∆**	***p*-Value**^**(W)**^		**Before**	**After**	**∆**	***p*- Value**^**(W)**^		
	Mean ± SD	Mean ± SD	Mean ± SD		**d**	Mean ± SD	Mean ± SD	Mean ± SD		**d**	
Inattentive	13.52 ± 7.3	7.86 ± 3.64	-5.14 ± 8	< 0.001*	0.74	12.2 ± 4.27	10.83 ± 3.9	-1.4 ± 3.73	0.042*	0.45	0.017*
Hyperactive/impulsive	12.8 ± 2.6	9.7 ± 3.5	-3.1 ± 3.7	< 0.001*	0.87	12.6 ± 3.7	11.4 ± 3.7	-1.2 ± 5.5	0.336	0.22	0.037*
Total	25.6 ± 4.4	19.6 ± 6.6	-6 ± 7.4	< 0.001*	0.85	25.7 ± 7.6	24.35 ± 7.8	-1.3 ± 10.4	0.561	0.13	0.034*
CPRS-R- (T score)											
Inattentive	62.6 ± 8	56 ± 7	-6.7 ± 10	0.001*	0.75	62 ± 8.4	59.6. ± 8.14	-2.5 ± 6.7	0.043*	0.45	0.007*
Hyperactive/impulsive	63.31 ± 6	56.53 ± 7	-6.8 ± 8.7	< 0.001*	0.85	62.8 ± 9.2	60.15 ± 7	-2.6 ± 12	0.218	0.28	0.035*
Total	63.6 ± 6.6	57.4 ± 3.2	-6.11 ± 8	< 0.001*	0.84	62.73 ± 7.2	60.3 ± 7	-2.5 ± 9.5	0.181	0.3	0.032*

*CPRS-R-L* Conners Parent Rating Scale–Revised Long Version

^(W)^Wilcoxon signed-rank test (Before vs. after comparison for each group separately); ∆ (After minus Before; ^(M)^ Mann–Whitney U-test (∆ comparison between the groups);*p < 0.05; d, The Cohen’s d effect size

**Table 3:** Child Behavioral Checklist, comparing the probiotic and control group results at baseline and follow-up measurements

CBCL Parent Form (Raw score)	**Probiotics *(N* = *36)***					**Control *(N* = *40)***					***p*- Value**^**(M)**^
	**Before**	**After**	**∆**	***p*-Value**^**(W)**^		**Before**	**After**	**∆**	***p*- Value**^**(W)**^		
	Mean ± SD	Mean ± SD	Mean ± SD		**d**	Mean ± SD	Mean ± SD	Mean ± SD		**d**	
Syndrome scale (Internalizing)	17 ± 1.3	14 ± 6	-3.1 ± 6.31	0.006*	0.62	16.5 ± 2.3	15.7 ± 2	-0.7 ± 2.7	0.064	0.41	0.007*
Syndrome scale (Externalizing)	18.14 ± 2.4	15.31 ± 3.6	-2.83 ± 4.5	0.001*	0.71	17.8 ± 3.4	17.2 ± 3	-0.6 ± 3	0.209	0.3	0.007*
Total Score	57.8 ± 3.4	53 ± 9.6	-5 ± 9.3	0.002*	0.70	58.8 ± 3.4	57.7 ± 3.5	-1.13 ± 4.23	0.072	0.40	0.007*
CBCL Parent Form (T score)											
Syndrome scale (Internalizing)	68.83 ± 2.3	66 ± 4.5	-2.8 ± 4.2	0.001*	0.74	68.5 ± 2	68 ± 2.2	-0.6 ± 2.7	0.055	0.43	0.014*
Syndrome scale (Externalizing)	69.6 ± 3.5	66.5 ± 4.3	-3.11 ± 4.7	< 0.001*	0.85	69.25 ± 3.3	68.6 ± 4	-0.67 ± 3.3	0.556	0.13	0.002*
Total Score	67.8 ± 6.6	62.7 ± 4.8	-5 ± 7.8	0.001*	0.71	68 ± 6	65.8 ± 6	-2.2 ± 8	0.0.82	0.40	0.024*

*CBCL* Child Behavioral Checklist

^(W)^Wilcoxon signed-rank test (Before vs. after comparison for each group separately), ∆ (After minus Before, ^(M)^ Mann–Whitney U-test (∆ comparison between the groups),*p < 0.05; d, The Cohen’s d effect size

Secondary outcomes.

Table 4 shows the CPT parameters of the children under study. Twelve weeks of intervention increased the correct responding rate during the entire number of trials (target accuracy rate) in the probiotics group compared with the control group with a significant mean change difference (*p* = 0.02). The absence of the required response (omission errors) for attention selectiveness was less in the probiotics group than in the control group, with a significant mean change difference between the two groups (*p* = 0.043). Although the probiotics group’s mean score decreased from baseline with a mean change over 12 weeks in reaction time (RT) on correct trials in ms (− 3.9 ± 96.56) and the standard deviation of RT (variability) (− 7.3 ± 134.7) compared with those of the control group (0.58 ± 130.13 and 1.31 ± 115.06, respectively), this difference did not reach statistical significance. Furthermore, the two groups did not differ in the mean and standard deviation of the error RT (*p* > 0.05). Addressing impulsivity, we found no significant difference between the two groups over 12 weeks in the inhibited RT during the entire number of trials (foil accuracy rate) (*p* = 0.606) and in the false required response (commission errors) (*p* = 0.559).

**Table 4:** Continuous Performance Test, comparing the probiotic and control group results at baseline and follow-up measurements

CPT	**Probiotics *(N* = *36)***						**Control *(N* = *40)***					***p*- Value**^**(M)**^
	**Before**		**After**	**∆**	***p*- Value**^**(W)**^		**Before**	**After**	**∆**	***p*- Value**^**(W)**^		
	Mean ± SD		Mean ± SD	Mean ± SD		**d**	Mean ± SD	Mean ± SD	Mean ± SD		**d**	
Correct trials		287.34 ± 20.57	293 ± 28.20	5.64 ± 13.6	0.014*	0.55	286.35 ± 33.9	288.37 ± 28.25	2.03 ± 13.24	0.459	0.17	0.094
Correct targets		275.64 ± 22.96	282.3 ± 29.78	6.66 ± 16.6	0.035*	0.47	275.9 ± 36.3	277.63 ± 29.18	1.7 ± 27.44	0.988	0	0.152
Correct foils		11.17 ± 5.65	13.1 ± 5.58	1.89 ± 4.86	0.039*	0.46	10.58 ± 4.03	11.35 ± 4.41	0.78 ± 4.18	0.419	0.18	0.062
Target Acc Rate		0.87 ± 0.08	0.90 ± 0.09	0.03 ± 0 .06	0.004*	0.65	0.84 ± 0.12	0.86 ± 0.09	0.01 ± 0.09	0.837	0.05	0.02*
Foil Acc Rate		0.30 ± 0.16	0.35 ± 0.17	0.05 ± 0.2	0.153	0.32	0.31 ± 0.13	0.35 ± 0.15	0.05 ± 0.15	0.161	0.31	0.606
Commission errors		25 ± 5.9	24.42 ± 8.23	-0.69 ± 7.5	0.426	0.18	25.48 ± 4.47	24.98 ± 4.69	-0.5 ± 4.98	0.525	0.14	0.559
Omission errors		46.88 ± 25.63	38.17 ± 26	-8.7 ± 16.3	0.004*	0.65	48.47 ± 36	46.55 ± 27.5	-1.93 ± 21.34	0.844	0.05	0.043*
Correct real time		534.75 ± 62.72	530.8 ± 73.02	-3.9 ± 96.56	0.652	0.10	531.58 ± 72.2	532.15 ± 128	0.58 ± 130.13	0.868	0.04	0.751
Correct RT SD		391.9 ± 155.83	384.6 ± 153.23	-7.3 ± 134.7	0.819	0.05	401.1 ± 144.4	402.4 ± 141.3	1.31 ± 115.06	0.814	0.05	0.673
Error RT mean		560.86 ± 171.79	545.8 ± 95.84	-15.03 ± 188.81	0.509	0.15	552.85 ± 90.73	543.45 ± 111.89	-9.4 ± 129.11	0.791	0.01	0.423
Error RT SD		440.68 ± 166.36	432.13 ± 165.99	-8.55 ± 257.38	0.789	0.06	434.02 ± 233.2	432.16 ± 238.44	-1.86 ± 280.6	0.829	0.05	0.992

*CPT* Continuous Performance Test, *Target Acc Rate* Target Accuracy Rate, *Foil Acc Rate* Foil Accuracy Rate, *Correct RT SD* Correct real-time standard deviation; Error RT mean, Error real-time mean; Error RT SD, Error real-time standard deviation

^W)^Wilcoxon signed-rank test (Before vs. after comparison for each group separately);∆ (After minus Before; ^(M)^ Mann–Whitney U-test (∆ comparison between the groups); *p < 0.05; The Cohen’s d effect size

Regarding WCST performance, a significant difference over 12 weeks was found in the probiotics group in the following indices: correct responses, perseverative responses, perseverative error (response when the old rule is still applied), and non-perseverative errors (attentional inability to inhibit distraction within the same perceptual category),(*p* = 0.016, *p* = 0.027, p = 0.017, and *p* = 0.044, respectively), but not in the control group. However, the Mann–Whitney U-test analysis of the mean change difference between the two groups in these indices revealed no significant differences (*p* > 0.05). The remaining index, including failure to maintain the set, did not significantly improve for either group (*p* > 0.05) (Table 5).

**Table 5:** Wisconsin Card Sort Test, comparing the probiotic and control group results at baseline and follow-up measurements

WCST	**Probiotics *(N* = *36)***					**Control *(N* = *40)***					***p*- Value**^**(M)**^
	**Before**	**After**	**∆**	***p*- Value**^**(W)**^		**Before**	**After**	**∆**	***p*- Value**^**(W)**^		
	Mean ± SD	Mean ± SD	Mean ± SD		**d**	Mean ± SD	Mean ± SD	Mean ± SD		**d**	
Wisconsin categories completed	3.67 ± 1.57	4.14 ± 1.91	0.47 ± 1.44	0.064	0.41	3.93 ± 1.97	4.33 ± 1.56	0.4 ± 1.81	0.143	0.33	0.499
Correct responses	83.97 ± 9.59	88.64 ± 10.41	4.67 ± 10.4	0.016*	0.54	81.95 ± 14.9	83 ± 13.1	1.1 ± 11.35	0.466	0.16	0.148
W total errors (%)	33.66 ± 7.75	31.29 ± 6.5	-2.36 ± 6.73	0.058	0.42	35.58 ± 11.79	34.65 ± 10.78	-0.93 ± 8.76	0.275	0.24	0.512
W perseverative responses (%)	34.63 ± 9.5	31.84 ± 7	-2.79 ± 7.31	0.027*	0.50	32.95 ± 11.34	31.68 ± 7.93	-1.3 ± 10.46	0.795	0.06	0.134
Perseverative error	17.88 ± 6.83	15.81 ± 5.1	-2.23 ± 6.27	0.017*	0.53	18.43 ± 6.16	17.55 ± 6.34	-0.88 ± 5.77	0.500	0.15	0128
Non-Perseverative Errors %	19.42 ± 12.94	16.33 ± 13.28	-3.1 ± 7.9	0.044*	0.45	17.55 ± 12.93	16.47 ± 10.2	-1.1 ± 7.73	0.434	0.17	0.242
Unique Errors? %	3.17 ± 1.93	2.52 ± 1.6	-0.66 ± 2.3	0.088	0.38	3.85 ± 2.74	3.63 ± 2.4	-0.22 ± 2.2	0.561	0.15	0.670
Trails to complete the first cat	26.88 ± 23.27	25.42 ± 24	-1.47 ± 9.84	0.294	0.23	25.3 ± 18.14	25.48 ± 19.14	0.18 ± 14.43	0.107	0.36	0.851
Failure to maintain set	3.64 ± 1.66	3.14 ± 1.6	-0.5 ± 1.8	0.156	0.32	3.33 ± 1.56	3.1 ± 1.82	-0.25 ± 2.2	0.539	0.14	0.483
Conceptional level response	54.78 ± 11.71	55.83 ± 12.93	1.1 ± 8.24	0.408	0.18	53.62 ± 12.1	52.5 ± 14.1	-1.12 ± 7.66	0.450	0.17	0.393

W total errors, Wisconsin total errors; Wisconsin perseverative responses; Trails to complete first cat, Trails to complete first category

^(W)^Wilcoxon signed-rank test (Before vs. after comparison for each group separately); ∆ (After minus Before; ^(M)^ Mann–Whitney U-test (∆ comparison between the groups; *p < 0.05; The Cohen’s d effect size

### Adverse events

An analysis of the medication’s potential adverse effects revealed no notable undesirable symptoms in either group. Seven participants (three in the probiotics group and four in the control group) reported decreased appetite. One patient from the probiotics group reported having diarrhea, which cleared up after a few days.

## Discussion

As knowledge of the GBA has risen with emerging research highlighting this bidirectional relationship with a theoretical translation of animal models to human analyses, the core mechanism and which probiotics have a promising or negative result remain ambiguous. To the best of our knowledge, this study is the first randomized controlled trial to use a *Lactobacillus acidophilus* LB strain as supplementation added to a weight-dependent dose of atomoxetine to examine its impact on the core symptoms, behavior, and cognition of children and adolescents with ADHD.

Twelve-week supplementation with L. acidophilus LB combined with a weight-dependent dose of atomoxetine could improve the symptoms and behavioral problems of ADHD, according to the parental reports of the CPRS-R-L and CBCL, respectively, relative to the control group.

Our findings support the results of a recent trial by Ghanaatgar et al. [32]. The study showed that taking a multispecies probiotic capsule containing 14 bacterial strains, including L. acidophilus, for 8 weeks alongside Ritalin medication positively affected the severity of ADHD symptoms. This was measured by improved scores on the Clinical Global Impression–Severity scale (CGI–S) and the Revised Conners Parent Rating Scale–Short Version (CPRS–RS).

Another study investigated the effects of probiotics on the psychological health of children with ADHD. The study found that probiotic treatment for 8 weeks, using four bacterial strains, including Lactobacillus acidophilus, significantly improved the severity of ADHD symptoms and anxiety compared to a placebo. The improvement was measured using the ADHD and Hamilton Anxiety Rating scales. However, probiotics did not have an impact on depression. [33]. Of note, a recent Taiwanese study found that using the oral probiotic *Bifidobacterium bifidum-688* (Bf-688) for 8 weeks reduced the clinical symptoms of patients with ADHD while increasing their body weights and body mass index. The Bf-688 supplement also markedly changed the composition of the gut flora [20].

Contradictory to our findings, Kumperscak et al. [34] evaluated the influence of LGG on treatment-naive children and adolescents with ADHD and found no considerable improvement in the core symptoms or mental health problems compared with those who received a placebo after 3 months. However, the authors found that the probiotic group significantly outperformed the placebo group in the Child Self-Report measure of quality of life and suggested including patients receiving stable pharmacotherapy in future trials to identify more significant changes. The fact that probiotic benefits can differ according to the strain employed, dose, duration, and methodological variations may help explain the variable outcomes between trials.

Available reviewing research has broadened our understanding of the link between probiotic supplementation and its impact on ADHD clinical symptoms. However, their findings remain inconclusive to formulate any clinical recommendation or approaches. As the underlying etiopathogenesis of ADHD is still unclear, investigating the role of the complex messaging system between the microbiota, gut, and brain has drawn much attention [35, 36]. Furthermore, researchers considered these pathways as an area that seems amenable to change in treating neurodevelopmental disorders, such as ADHD, possibly without adverse effects.

Considering that the GBA hypothesis has been linked to the pathophysiological pathways underlying ADHD [36], it may be reasonable to view probiotic supplementation's ability to address gut dysbiosis as a possible treatment target for ADHD. Moreover, probiotics' effectiveness in treating ADHD symptoms may be due to their ability to prevent an inflammatory reaction [37]. A microbial imbalance is associated with a breakdown of the immune system’s homeostasis by a boost in potentially inflammatory bacteria, which disrupts intestinal permeability and can increase the movement of pathogenic bacteria’s metabolites into the systemic circulation, which may lead to an inflammatory process [12, 13]. Hence, this may affect the permeability of the blood–brain barrier, contributing to neuroinflammation in neurodevelopmental disorders such as ADHD [14, 15]. Probiotics can improve gut integrity, preventing metabolite leakage and inhibiting the inflammatory cascade [37, 38].

Regarding the secondary outcomes under study, our current analysis found that the probiotic group revealed improvement in the target accuracy rate and omission errors compared with the control group (medium effect size), suggesting that *Lactobacillus acidophilus* LB could have a favorable effect on focused attention as indicated by the CPT. However, we did not find a comparable improvement in impulsivity.

Mounting evidence suggests that the cholinergic and dopaminergic systems contribute to the pathophysiology of selective attention [39]. An interesting study found that the psychobiotic strain Lactobacillus PS128 improved CPT measures of ADHD in children with Tourette syndrome [40]. The authors attributed the improvements to PS128’s potential to regulate serotonergic and dopaminergic signaling in mouse brains, as demonstrated by experimental findings from animal models [41].

Our results showed improvement in the executive functions in the probiotic group reflected in both perseverative and non-perseverative errors on WCST performance (small-medium effect size); however, this did not reach a significant level of differences between the two groups.

Impairments in executive functions (i.e., cognitive flexibility, working memory, sustained attention, inhibitory control, and planning) are considered a core deficit in the cognitive function of ADHD, which may play a crucial role in the challenging adaptation of ADHD [42, 43].

The cognitive regulation of behavior and reward perception is modulated via the norepinephrine and dopamine circuits, which connect to the prefrontal cortex and striatum, and these pathways are considered fundamental in the pathophysiology of ADHD [44].

Until now, limited randomized trials have investigated the impact of probiotic supplementation on cognitive performance [45]. Although a questionable probiotic strain influences cognition, one study employing *Lactobacillus rhamnosus* supplements demonstrated a favorable influence on cognitive function and a lowered risk of developing ADHD [18].

The linkage between ADHD and the microbiota can be understood in terms of how neurotransmitters function in cognition. A recent study has provided insights into the GBA and introduced a new strain that improves cognitive function through this axis. Using healthy mice, Jeon et al. [46] investigated the effects of three probiotic groups on cognitive function: *Lactobacillus acidophilus* EG004, *Lacticaseibacillus rhamnosus*, and *Lacticaseibacillus paracasei*. The three probiotic-fed testing groups demonstrated better cognitive function; however, the *L. acidophilus*-fed group was superior to the other two groups and scored the highest on cognitive–behavioral assessments. Focusing on understanding how the changed microbial diversity affects the brain, a 16S-23S rRNA sequencing of the gut microbiome of the *L. acidophilus* group was performed. It was found that the *L. acidophilus* group had an elevated proportion of *L. acidophilus* presence, suggesting that a good proportion of *L. acidophilus* can be adequately ingested without being harmed by the digestive juices. Researchers hypothesized that an increase in *L. acidophilus* in the intestines modifies neurotransmitters and neurotrophic factors, including dopamine, noradrenaline, gamma-aminobutyric acid (GABA), and serotonin, affecting an animal’s nervous system. Interestingly, they found that ingesting *L. acidophilus* increased SCFAs in the gut of experimental mice, indicating *L. acidophilus*’s ability to produce SCFAs, which may positively impact brain function. The microbially fermented compounds SCFAs, such as acetate, propionate, and butyrate, stimulate indirect signaling in the brain by modifying and inducing neurotransmitters and neurotrophic factors, such as GABA and BDNF [47, 48]. Considering this information, the authors reported the inability to fully identify the changed metabolites from the animal body, which is required to understand the mechanism underlying the improved cognitive ability.

In the present study, most participants were males. The finding aligns with previous studies indicating that ADHD is more common in boys [49, 50]. However, evidence displayed a comparable clinical profile in boys and girls [51, 52].

### Limitations

This study is an open-label study without a placebo intervention. Given that the parents were knowledgeable of the treatment their child had experienced, this potentially may have affected how they responded to the questionnaires. However, parallel to the parent responses, we also included various objective measure evaluations (i.e., the CPT and WCST), and the results showed the advantage of adding probiotics to the standard treatment alone. While our findings showed that a 3-month probiotic intervention was beneficial, there were no observable changes in some cognitive functions measured by the neuropsychological assessment battery, leading us to believe that the study’s duration was insufficient to track the improvements. Therefore, further research with a longer intervention time is needed. In this study, we did not investigate the socioeconomic status of the groups; however, SES should be assessed in microbiome studies, given that it can be an influential confounding variable that impacts the analysis of the study results [53]. Finally, the homogeneity of the sample’s ethnic and geographic distribution, its small size, and its recruitment from two similar referral centers may restrict the generalizability of our findings.

## Conclusion

In conclusion, this study has demonstrated that 3 months’ supplementation of oral probiotics, such as *Lactobacillus acidophilus* LB (Lacteol Fort) added to a weight-dependent dose of atomoxetine improved the severity of symptoms, sustained attention, and executive functions in children and adolescents with ADHD. Considering this information, *Lactobacillus acidophilus* LB may be a desirable supplementary therapy for children with ADHD without side effects. Future research is recommended to verify the treatment impacts of the probiotic *Lactobacillus acidophilus* LB on the core symptoms and cognitive functions of ADHD.

## Data Availability

All data generated or analyzed during this study are included in this published article or are available from the corresponding author on reasonable request.

## Abbreviations

ADHD: Attention-deficit/hyperactivity disorder
BDNF: Brain-derived neurotrophic factor
Bf-688: Bifidobacterium bifidum-688
CBCL: Child Behavioral Checklist
CPRS-R-L: Conners Parent Rating Scale–Revised Long Version
CPT: Conners Continuous Performance
GBA: Gut–brain axis
SCFAs: Short-chain fatty acids
HPA: Hypothalamic–pituitary–adrenal
LGG: Lactobacillus rhamnosus GG
L. acidophilus: Lactobacillus acidophilus
PEBL: Psychology Experiment Building Language
WISC-III: Wechsler Intelligence Scale for children
WCST: Wisconsin (Berg) Card Sort Test
